# Diel changes and diversity of *puf*M expression in freshwater communities of anoxygenic phototrophic bacteria

**DOI:** 10.1038/s41598-019-55210-x

**Published:** 2019-12-10

**Authors:** Lívia Kolesár Fecskeová, Kasia Piwosz, Martina Hanusová, Jiří Nedoma, Petr Znachor, Michal Koblížek

**Affiliations:** 1 Lab. of Anoxygenic Phototrophs, Centre Algatech, Institute of Microbiology of the Czech Academy of Sciences, 37981 Třeboň, Czech Republic; 2 Biology Centre of the Czech Academy of Sciences, Institute of Hydrobiology Na Sádkách 702/7, 370 05, České Budějovice, Czech Republic

**Keywords:** Microbiology, Environmental sciences, Limnology

## Abstract

The anoxygenic phototrophic bacteria (APB) are an active component of aquatic microbial communities. While DNA-based studies have delivered a detailed picture of APB diversity, they cannot provide any information on the activity of individual species. Therefore, we focused on the expression of a photosynthetic gene by APB communities in two freshwater lakes (Cep lake and the Římov Reservoir) in the Czech Republic. First, we analyzed expression levels of *puf*M during the diel cycle using RT-qPCR. The transcription underwent a strong diel cycle and was inhibited during the day in both lakes. Then, we compared DNA- (total) and RNA-based (active) community composition by sequencing *puf*M amplicon libraries. We observed large differences in expression activity among different APB phylogroups. While the total APB community in the Římov Reservoir was dominated by Betaproteobacteria, Alphaproteobacteria prevailed in the active library. A different situation was encountered in the oligotrophic lake Cep where Betaproteobacteria (order Burkholderiales) dominated both the DNA and RNA libraries. Interestingly, in Cep lake we found smaller amounts of highly active uncultured phototrophic Chloroflexi, as well as phototrophic Gemmatimonadetes. Despite the large diversity of APB communities, light repression of *puf*M expression seems to be a common feature of all aerobic APB present in the studied lakes.

## Introduction

Anoxygenic phototrophic bacteria (APB) are prokaryotic organisms that capture light energy using bacteriochlorophyll (BChl)-containing reaction centers^1^. Upon illumination, the photosynthetic reaction centers drive electron transport and pump protons across the membrane. The reducing equivalents and proton gradient are subsequently utilized in the cellular metabolism. There are two types of photosynthetic reaction centers^2^. The Type-1 reaction centers (FeS-based) are found in phototrophic Chlorobi, Firmicutes, and Acidobacteria. Type-2 reaction centers (pheophytin-quinone type) are found in the phototrophic members of Proteobacteria, Chloroflexi, Gemmatimonadetes^3^ and in an uncultured candidate phylum WPS-2^4^.

The first cultured APB species were anaerobic Proteobacteria (representatives of current genera *Chromatium*, *Rhodospirillum* and *Rhodobacter*), and Chlorobi^5^. These organisms express their photosynthetic genes and conduct photosynthesis under anaerobic conditions, and their pigment synthesis is repressed by oxygen^6^. The same applies to the first discovered phototrophic Chloroflexi, which thrive mostly under anoxic conditions^7^. These initial discoveries about APB had led to the long-lasting impression that APB typically live and photosynthesize under anaerobic conditions.

This paradigm started to change in late 1970’s when several aerobic BChl-containing strains were isolated from coastal environments in Japan^8^. In contrast to classical anaerobic APB, these organisms grew, metabolized and synthesized BChl under fully oxic conditions^9^. These species are called Aerobic Anoxygenic Phototrophic (AAP) bacteria, and are common in many natural habitats. They represent 1–10% of total bacteria in the euphotic zone of the ocean contributing significantly to the secondary carbon production^10^, reviewed by Koblížek^11^. They are also common in freshwaters, where they represent up to 37% of total bacteria^12–14^.

Diversity of the APB, both aerobic and anaerobic, is often studied using specific marker genes associated with anoxygenic photosynthesis^15–17^. The most common marker of APB containing Type-2 reaction centers (Proteobacteria, Gemmatimonadetes, Chloroflexi and WPS-2 species^3,4^) is the *puf*M gene, which encodes the M subunit of bacterial reaction centers^18,19^. Diversity of the *puf*M gene was investigated in various aquatic habitats, including open ocean^20,21^, estuaries^22^, coastal lagoons^23^, permanently frozen lakes^24^, saline lakes^25^ and freshwater lakes^26,27^. Presently, next-generation sequencing technologies enabled to explore *puf*M diversity to an unprecedented degree^16,28,29^.

While diversity studies have delivered a detailed picture of APB communities, they do not provide any information on photosynthetic activity by individual phylotypes. The sole presence of the genes does not guarantee corresponding activity in an environment. For instance, numerous species of an abundant freshwater genus *Limnohabitans* were found to contain photosynthetic genes, but the phototrophic phenotype has been reported in only one strain^30^. This highlights the importance of examining not just abundance or presence of the functional genes, but also their expression. In contrast to a large number of environmental DNA-based diversity studies, data on *in situ* photosynthetic gene expression is scarce. Currently, only available information originates from a few metatranscriptomic studies. An analysis of marine bacterial community collected at the station ALOHA (Hawaii), showed that genes associated with anoxygenic phototrophy (*puf, bch*) were expressed amongst the highest, despite their gene abundance in the corresponding DNA library being rather low^31^. In contrast, Sieradzki *et al*.^32^ have reported very low expression of genes related to anoxygenic phototrophy in their seasonal survey of gene expression in the San Pedro Channel. Even less information is available from freshwater environments. Vila-Costa *et al*.^33^ reported that in a mountain lake Llebreta, Spain, the photosynthetic genes (chlorophyll-, proteorhodopsin- and BChl-related) were transcribed mostly during the daylight hours.

To address these contradicting results, we measured diel changes in expression of the *puf*M gene by natural APB communities in two freshwater lakes in the Czech Republic. We were interested in (i) which freshwater APB species express their *puf*M genes in the fully aerobic euphotic zone, (ii) how is the expression influenced by light, (iii) how the individual APB species differ in their expression activity. The expression of the *puf*M gene was followed using RT-qPCR and the composition of total and active APB communities was analyzed using DNA and RNA *puf*M amplicon libraries, respectively.

## Results

### Environmental conditions

#### Cep lake

**Figure 1:**
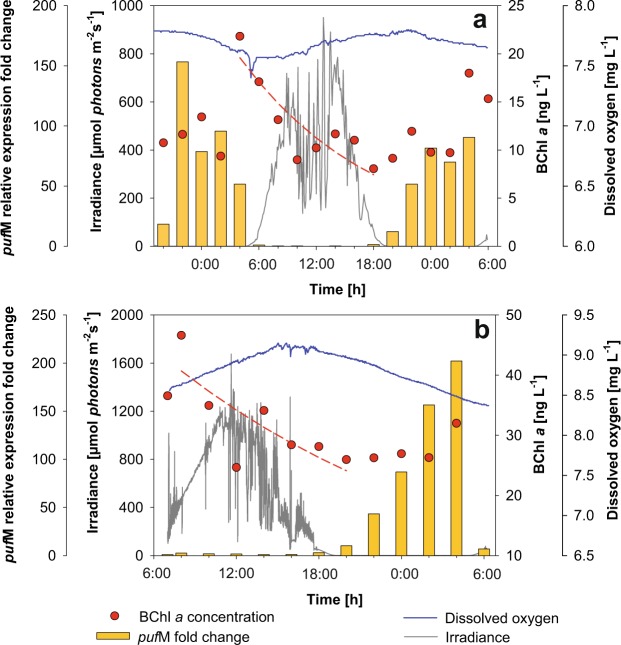
Under water irradiance, concentrations of bacteriochlorophyll *a* (BChl *a*), concentrations of dissolved oxygen and *puf*M relative expression fold change in Cep lake (**a**); and the Římov Reservoir mesocosm (**b**).

**Figure 2:**
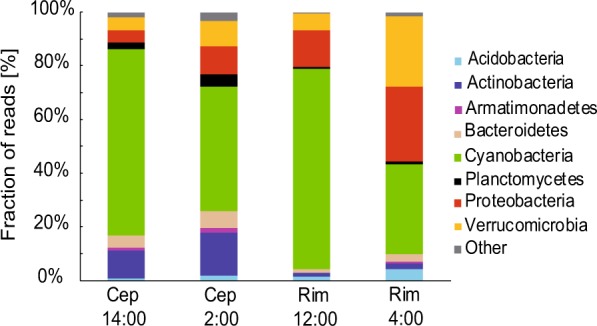
Relative abundance of the main phyla in the 16S rRNA transcript amplicon library of Cep lake (Cep) and the Římov Reservoir (Rim) in day and night samples.

The first experiment was conducted at Cep lake in August 2016. The general conditions were typical for an oligotrophic lake in summer, with stratified water column and fully oxygeneted surface layer (Fig. 1a). We registered changes of main physicochemical and biological parameters including temperature, pH, oxygen concentration, Chl *a*, and total and APB bacterial abundance during the diel cycle (Table 1). The active prokaryotic community was typical for the season with Cyanobacteria (mostly *Cyanobium* spp.), Actinobacteria, Verrucomicrobia, Bacteroidetes and Proteobacteria (mostly Alpha- and Deltaproteobacteria) and lower contribution of Planctomycetes, Armatimonadetes and Acidobacteria (Fig. 2, Supplementary Fig. S1). To evaluate the impact of a light/dark cycle on the active prokaryotic community we compared 16S rRNA transcript libraries. As expected, the proportion of Cyanobacteria largely differed between day and night samples: at night (2:00) Cyanobacteria represented 43% of the library, but increased up to nearly 70% during the day (14:00) (Fig. 2).

**Table 1:** Characteristics of the lakes and physico-chemical and biological conditions during the sampling campaigns.

	Cep lake	Římov Reservoir
Latitude, longitude	48.94°N 14.88°E	48.85°N 14.49°E
Altitude (m)	450	471
Area (ha)	130	210
Depth (m)	12/10*	43/16.5**
Temperature (°C)	21.3–22.1	19.7–21.2
pH	8.2–8.3	9.6–9.7
Total phosphorous (µg L^−1^)	10.7	19.5
DOC (mg L^−1^)	3.82	7.65
Dissolved oxygen (mg L^−1^)	7.4–7.8	8.4–9.2
Chlorophyll (µg L^−1^)	2.0–4.1	15.6–23.1
BChl *a* (ng L^−1^)	8.1–21.8	24.7–46.6
Thymidine uptake (pmol L^−1^ h^-1^)	27–32	18–110
Total bacteria (10^6^ cells mL^−1^)	2.25–4.66	2.19–3.97
APB bacteria (10^5^ cells mL^−1^)	0.74–3.51	1.28–2.96
Percentage of APB (avrg.)	5.97 ± 1.73%	7.11 ± 1.34%

^*^Maximum depth/depth at the sampling point

^**^Maximum depth/average depth.

#### Římov Reservoir

The second experiment was conducted at the Římov Reservoir in August 2017. Phytoplankton biomass was concentrated in the upper (0–4 m) layer, which corresponded to the depth of epilimnion (Supplementary Fig. S2). During the experiment, phytoplankton assemblage was dominated by desmids (*Staurastum planktonicum*) with a minor portion of Cyanobacteria, Cryptophytes and green algae (Supplementary Fig. S3). The major prokaryotic phyla identified here were Cyanobacteria (dominated by genus *Microcystis*), Proteobacteria (mainly Alphaproteobacteria, dominated by the *Roseomonas* genus), Verrucomicrobia, Acidobacteria, Bacteroidetes, Actinobacteria and Planctomycetes (Fig. 2, Supplementary Fig. S1). Here, Cyanobacteria contributed with 33% to the night (4:00) 16S rRNA library, whereas during the day (12:00) their proportion increased up to 75% (Fig. 2). We did not observe such large diurnal changes in any other bacterial phyla (Supplementary Fig. S1).

Total bacterial abundance, determined by microscopy counts, had its minimum in the morning, increased during daylight hours reaching its maximum in the afternoon and declining during the night (Supplementary Fig. S4). Thymidine incorporation by total bacterial community followed the pattern of bacterial numbers with maximum at 18:00, which coincided with the maximum rate of primary production measured between 14:00–18:00 (Supplementary Fig. S4).

### Diel changes in APB community and *puf*M expression

Large diel changes were observed also in the APB community. On average, APB represented 6.0 ± 1.7% of total bacteria in Cep lake. Their abundance varied during the day with the lowest numbers (0.735 × 10^5^ cells mL^−1^) observed in the morning and the highest (3.51 × 10^5^ cells mL^−1^) in the afternoon. In contrast, BChl *a* concentration decreased during the day and recovered during the night, reaching its maximum in the early morning (Fig. 1a). The decay rate of BChl *a* during daylight hours, a value that corresponds to APB mortality, was 1.66 ± 0.38 d^−1^.

A strong diel pattern of APB abundance was observed also in the Římov Reservoir (Supplementary Fig. S4). Here, APB represented 7.11 ± 1.34% of total bacteria. Their numbers increased throughout the day reaching the maximum (2.96 × 10^5^ cells mL^−1^) at 16:00, after which the numbers decreased to minimum (1.28 × 10^5^ cells mL^−1^) during the night (Supplementary Fig. S4). BChl *a* reached maximum concentration in the morning and decreased to minimum in the evening (Fig. 1b). During the daylight hours the BChl *a* decay rate was 1.37 ± 0.16 d^−1^.

In previous studies, it was suggested that the decline of BChl *a* concentration was caused by the inhibition of *de novo* BChl *a* synthesis by light^34,35^. This has been documented under laboratory conditions^36^, however, it cannot be assumed that the same mechanism is present in a whole community of APB. BChl molecules are bound to the reaction center proteins, encoded by the *puf* genes. So, BChl and PufM protein synthesis occurs always together. Therefore, we followed the expression of the *puf*M gene through the diel cycle. We found that *puf*M expression underwent a strong diel cycle and was expressed only during the night in both lakes (Fig. 1). While the expression was stopped almost immediately after sunrise, its recovery after sunset took several hours.

### Diversity and composition of total and active APB communities

To find out which APB species contributed to the *puf*M expression, we performed *puf*M amplicon sequencing from DNA and RNA templates, for total and active APB community composition. RNA amplicons were generated from night RNA samples collected at 2:00 (Cep) or at 4:00 (Římov Reservoir), as the daylight RNA samples yielded no *puf*M PCR products. Since the experiment at Cep lake spanned two nights, two RNA amplicon libraries were generated, referred to as RNA1 and RNA2. In total, over 400,000 sequences obtained from both lakes were clustered at 94% similarity into 779 OTUs (1 519 OTUs with singletons). For further analyses this dataset was reduced to 392 OTUs (OTUs containing ≥ 10 sequences, see Supplementary Data Set). Rarefaction curves of rarefied *puf*M libraries indicate that APB diversity was well covered in both lakes (Supplementary Fig. S5). In general, alpha diversity was similar in both lakes and between the DNA and RNA libraries (Supplementary Table S2).

We have to note that taxonomic affiliations were greatly hindered due to the relatively short *puf*M amplicon (140 bp without primers) and paucity of reference sequences. OTU taxonomy affiliations were assessed based on the best blast hit to reference sequences in the GenBank. Average OTU similarity to reference sequences was 86.3% and average coverage 97%. All similarity values to the best blast hit and alignment coverage are given in the Supplementary Data Set. Often, due to the limited length of the amplicon and low similarity values, it was impossible to rigorously identify the OTU’s taxonomy below the order level. Therefore, all the taxonomy was assigned only at the level of order.

At the phylum level, the majority of the identified sequences were affiliated with Proteobacteria (78–98%) in both lakes and a smaller portion of sequences represented phototrophic Gemmatimonadetes and Chloroflexi. Despite the apparent similarity at the phylum level, the two lakes differed largely at the phylotype level. Interestingly, only less than 10% of the identified OTUs were present in both lakes, indicating that the studied lakes were inhabited by distinct phototrophic communities.

#### Cep lake

**Figure 3:**
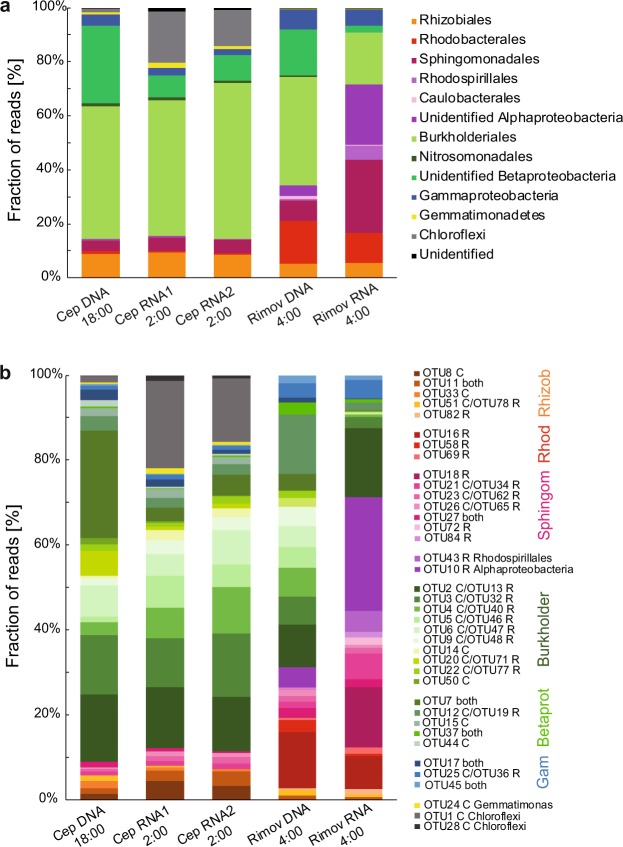
APB total (DNA-based) and active (RNA-based) community composition based on *puf*M amplicon library from Cep lake and the Římov Reservoir mesocosm during the diel experiment. *puf*M libraries were created from samples collected at indicated times. Community composition at the order level (**a**) and OTU level (50 most abundant OTUs) (**b**). The presence of a given OTU in lake library is indicated in the legend next to OTU number (C = OTU present only in Cep, R = present only in Římov, or in both lakes.) RNA1 and RNA2 refer to RNA collected on the first and second night of the sampling campaign at Cep lake.

The majority of *puf*M sequences in the DNA library (78% of total sequences, 120 OTUs) were affiliated with Betaproteobacteria, mostly (65%) belonging to Burkholderiales (Fig. 3a). Majority of these OTUs showed the highest similarity to *Limnohabitans* (40% of Betaproteobacteria). In total, 36% of Betaproteobacteria (28.7% of total) could not be identified to lower taxonomic levels. The second most abundant group was Alphaproteobacteria representing 14.5% of total sequences in the DNA library. Despite the lower contribution, Alphaproteobacteria were relatively diverse with 93 OTUs belonging to orders Rhizobiales (60% of Alphaproteobacteria), Sphingomonadales (27%), Rhodobacterales (8.7%) and Rhodospirillales (1.8%), and unidentified Alphaproteobacteria (1.7%) (Fig. 3a). In addition, we identified 4% of sequences affiliated with Gammaproteobacteria. Finally, we identified a small fraction of sequences related to phototrophic Chloroflexi and Gemmatimonadetes, both representing about 1% of the total sequences.

**Figure 4:**
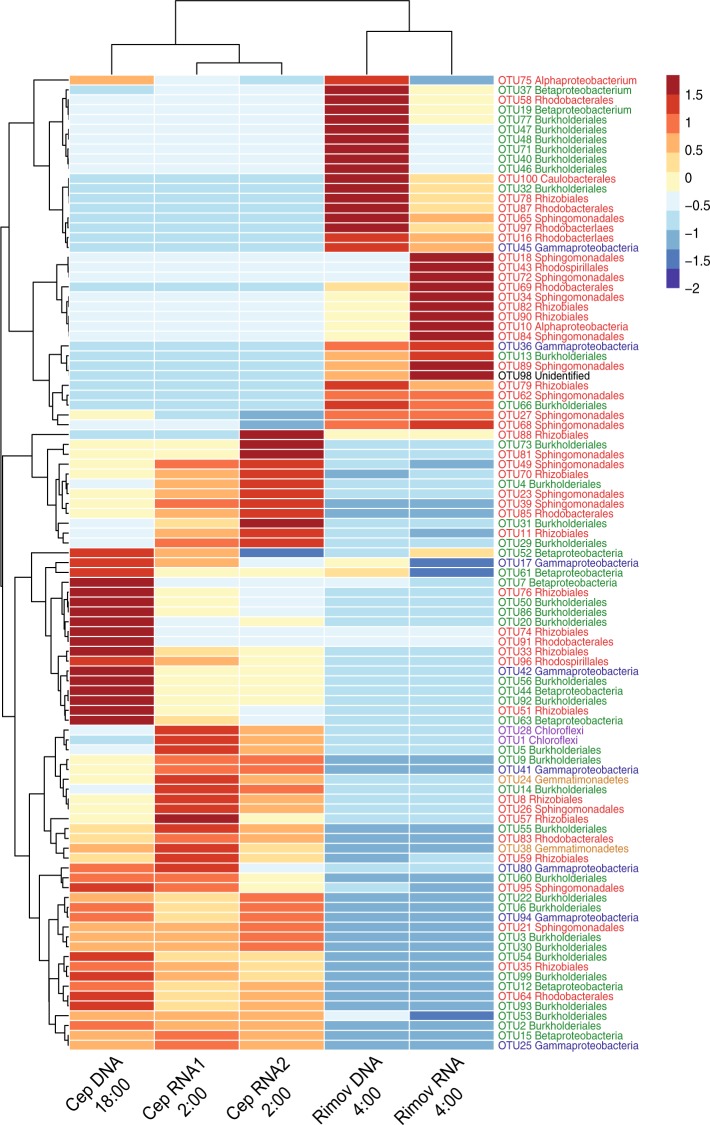
Heatmap of 100 most abundant *puf*M OTUs showing differences between total (DNA-based) and active (RNA-based) APB communities in Cep lake and the Římov Reservoir mesocosm. Blue color represents low contribution and red color represents high contribution of an OTU. Clustering was done using unweighted pair group method with arithmetic mean (UPGMA) method on Bray-Curtis distances calculated from percent data. The values were centered and scaled by removing the mean and then dividing by the standard deviation to facilitate visualization of both abundant and rare OTUs.

A somewhat different pattern was obtained when RNA amplicon library was analyzed. Similar to the DNA library, the active APB community was dominated by Betaproteobacteria in this lake (Fig. 3). Also, the contribution of Alphaproteobacteria to the RNA library mostly reflected their contribution to the DNA library (Fig. 3). Gammaproteobacterial OTUs (OTU17 and OTU42) were less abundant in the RNA library compared to DNA (Figs. 3b and 4). Interestingly, non-proteobacterial phyla contributed much more to the active fraction of the APB community than to its total composition. Phototrophic Gemmatimonadetes exhibited a higher contribution to the RNA library than to DNA (OTU24) (Figs. 3b and 4). However, the most striking result was obtained for OTU1 and OTU28 affiliated with Chloroflexi. Despite of its low (<1%) abundance in DNA, OTU1 represented the most abundant OTU in the RNA library, with 18% of all the sequences (Figs. 3b and 4). Also less abundant OTU28 was approx. 10 times more abundant in the RNA library than in the DNA (Fig. 4).

**Figure 5:**
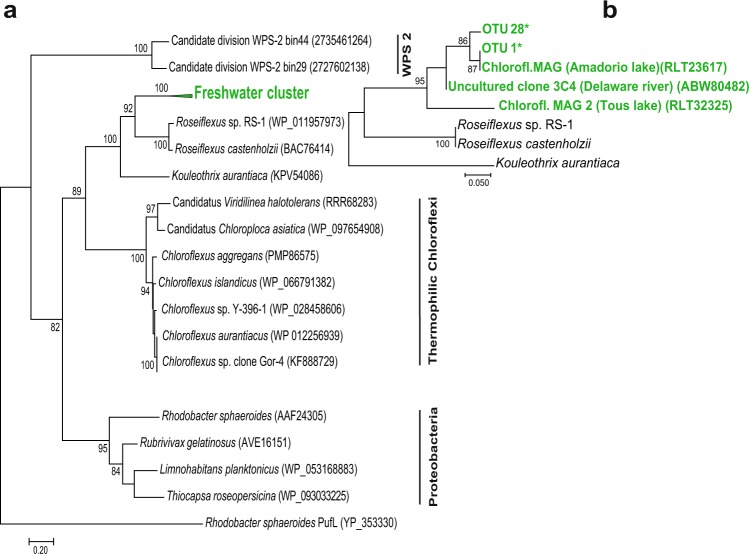
PufM protein Maximum Likelihood phylogenetic tree, computed using the LG substitution model. A discrete Gamma distribution was used to model evolutionary rate differences with invariant sites. The analysis involved 20 amino acid sequences and a total of 297 positions (**a**), respectively 25 amino acid sequences and a total of 48 positions (**b**) in the final dataset. Bootstrap values > 70 are shown. *Rhodobacter sphaeroides* PufL sequence was used as an outgroup.

Interestingly, the obtained Chloroflexi phylotypes OTU1 and OTU28 did not match with any cultured species in the GenBank database. Thus, we constructed PufM protein phylogenetic tree using cultured species and environmental sequences. OTU1 and OTU28 clustered closely with a few, recently reported Chloroflexi PufM sequences from freshwater environments: MAGs from lakes Tous and Amadorio in Spain^37^ and clones from Delaware River in USA^22^ (Fig. 5). In the phylogenetic tree, sequences of uncultured freshwater Chloroflexi formed a distinct, well supported branch (bootstrap = 92) within the *Kouleothrix* cluster.

#### Římov Reservoir

Similar to the situation in Cep lake, the majority of *puf*M sequences in the DNA library of the Římov Reservoir were represented by Betaproteobacteria (57% of sequences, 54 OTUs). Here, as well as in Cep, Burkholderiales was the main order (60% of Betaproteobacteria) with most of the OTUs related to *Limnohabitans* (39% of Burkholderiales). Approx. 30% of Betaproteobacteria remained unidentified. In contrast to Cep lake, there was a much higher proportion of Alphaproteobacteria (33.7% of sequences and 67 OTUs). These OTUs were affiliated with orders Rhodobacterales, Sphingomonadales, Rhizobiales, Caulobacterales and Rhodospirillales, representing 46%, 22%, 15.5%, 3% and 1.2% of Alphaproteobacteria, respectively (Fig. 3a). Over 10% of alphaproteobacterial sequences could not be further identified. Gammaproteobacteria were found to be more abundant (7.5%) compared to Cep lake, while phototrophic Chloroflexi and Gemmatimonadetes were only minor groups with 0.05% resp. 0.14% of all the sequences.

In contrast to the DNA library, the active fraction of the Římov Reservoir was dominated by Alphaproteobacteria (70% of sequences in the RNA library), mostly by members of Sphingomonadales or unidentified Alphaproteobacteria (Fig. 3). Many alphaproteobacterial OTUs were 10-times more represented in the RNA than in the DNA library (Figs. 3b and 4). The most active were the OTUs showing the highest similarity to *Sphingomonas* (OTU18, OTU34), *Porphyrobacter* (OTU72, OTU84, OTU190), *Roseococcus* (OTU43), *Hyphomonadacea* (OTU69) and to unidentified Alphaproteobacteria (OTU10) (Fig. 4). Betaproteobacteria were substantially less active (Fig. 3), and many OTUs were represented in the RNA library 10 times less when compared to the DNA library, or were even absent. Examples include *Polynucleobacter*-related OTUs (OTU40, OTU46), many *Limnohabitans*-related OTUs (OTU47, OTU48, OTU77, OTU110) and unidentified Betaproteobacteria (OTU37, OTU61) (Fig. 4) This indicates that most of the present phototrophic Betaproteobacteria were not actively expressing *puf*M genes in this particular environmental situation. Phototrophic Chloroflexi and Gemmatimonadetes represented 0.02% and 0.22% of the sequences, respectively.

## Discussion

One of the main results of this study is the inhibition of *puf*M expression during daylight hours in natural freshwater environments. The light repression of BChl *a* synthesis has been reported previously in the marine APB *Roseobacter denitrificans* grown under aerobic conditions^38^. The same repression of BChl *a* synthesis was also shown in freshwater aerobic APB *Erythromicrobium hydrolyticum*^39^. Later, it was shown that light exposure of *Roseobacter denitrificans* caused a repression of *puf* transcription^40^. BChl synthesis and *puf*M gene expression are tightly linked processes. Pigment molecules are bound to the reaction center proteins (*puf* gene products) with fixed stoichiometric ratios^41^. Therefore, the synthesis of BChl molecules and reaction center proteins runs together. In a more recent study of transcriptome dynamics in light-dark grown *Dinoroseobacter shibae*, it was shown that light exposure led to a rapid repression of the entire photosynthesis gene cluster^36^.

Our results extend the previous observations from bacterial cultures to complex freshwater APB communities (Fig. 1). It seems that freshwater APB species share the same regulation by light, which inhibits *puf*M transcription during light exposure. Interestingly, the same regulation is probably also present in phototrophic Chloroflexi that belong to a different phylum than phototrophic Proteobacteria. The fact that the same expression pattern was observed in two lakes with different trophic status and distinct composition of total bacterial and APB communities, documents that the observed regulation is widespread (if not universal) among APB living under aerobic conditions.

Naturally, APB had to develop complex regulatory mechanisms for their photosynthetic genes, with light being the main environmental factor affecting all phototrophs^42^. It seems that all the APB try to avoid the simultaneous presence of light and oxygen during BChl synthesis, as this may lead to the generation of harmful reactive oxygen species^40^. Anaerobic APB, such as the purple non-sulfur photosynthetic bacteria, downregulate the expression of the photosynthetic apparatus in the presence of light; however, the expression is usually not completely inhibited^42^. In addition, anaerobic APB developed the oxygen repression system, which shuts down the expression in the presence of oxygen^6^. Aerobic APB species (such as AAP bacteria) do not have this option. Given the permanent presence of oxygen in their environment, these organisms had to develop a regulatory mechanism that restricts BChl synthesis to dark periods.

It is very likely that light repression of *puf*M transcription also occurs in marine environments, where the inhibition of BChl *a* synthesis during the day was reported earlier^34^. This may explain the previously mentioned (see Introduction) contradictory reports from marine metatranscriptome studies. While the San Pedro Channel was sampled during the day and resulted in very low expression activity of the APB genes^32^, the samples at the station ALOHA (Hawaii) were collected before sunrise, and the *puf* genes were among the most actively expressed^31^. Yet, our findings contradict the results of a diel study from an oligotrophic mountain lake Llebreta, reporting expression of BChl-related genes during the day^33^. That study was based on only four samples, of which only one sample was collected during the day-light hours. Concerning fast changes in photosynthetic gene expression upon change of light conditions (Fig. 1)^36^, more frequent sampling and immediate processing of the water for RNA is absolutely crucial. Further research is necessary to verify whether expression of photosynthetic genes is differentialy regulated in mountain lakes. It is to be noted that a recent metatranscriptomic diel study conducted in Wisconsin lakes, USA, confirmed our results reporting that *puf*M gene expression peaked at night^43^.

The second part of this study focused on the diversity of local phototrophic communities. In general, APB communities in both lakes were dominated by Alpha- and Betaproteobacteria (Fig. 3), which is consistent with previous reports^16,26,27,44,45^. However, these two lakes differed largely at the OTU level, with only less than 10% of the OTUs shared between them (Fig. 4). In Cep lake, most of the phylotypes contributed similarly to both DNA and RNA libraries (Figs. 3 and 4), indicating widespread phototrophic energy acquisition in this oligotrophic environment. In contrast to Cep lake, the differences between the total APB community and its active fraction in the Římov Reservoir were conspicuous already at the class level, with Betaproteobacteria dominating the DNA library and Alphaproteobacteria prevailing in the RNA library (Fig. 3). In both lakes the total APB community was dominated by Burkholderiales order of the Betaproteobacteria, especially OTUs related to *Limnohabitans* emerged as the most abundant phylotypes (representing in total 15% of OTUs and 26.5% of all sequences from both lakes). This genus is recognized as one of the key freshwater bacterioplankton groups with a globally ubiquitous distribution^46,47^. Initially, the genus was thought to contain only heterotrophic species, and its phototrophic potential had been recognized only upon genome sequencing^48^. Even though the average OTU similarity to *Limnohabitans* sequences was 85%, the representative sequence of OTU207, present in both lakes and active in Cep lake, was identical over the full amplicon sequence to type strain *L. planktonicus* II-D5 (Supplementary Data Set). This provides the first evidence on their active phototrophy under *in situ* conditions in freshwater environments.

An unexpected discovery was the high expression activity of Chloroflexi-related *pufM* genes (almost 20%). To our knowledge phototrophic expression activity of Chloroflexi in freshwaters has not been reported before. The first cultured Chloroflexi were thermophilic species originating from various thermal springs^7,49,50^. In recent years, several studies registered *puf*M sequences belonging to phototrophic Chloroflexi in various freshwater habitats^16,22,37^. Here, we provide further evidence that freshwater phototrophic Chloroflexi were not only present, but also highly active in photosynthetic gene expression.

Another interesting result is the presence of phototrophic Gemmatimonadetes in both DNA and RNA libraries in both lakes (Figs. 3 and 4). Gemmatimonadetes were originally considered mostly soil-dwelling organisms^51,52^, and only recently they were also detected in pelagic environments^16,53^. Our findings indicate that phototrophic Gemmatimonadetes are not passively brought to the water column from sediment or soil, but that they are an active part of planktonic APB communities.

In contrast to the oligotrophic Cep lake, the environment of Římov Reservoir seemed to be more favorable for alphaproteobacterial phototrophy. Here, the observed high expression was originating mainly from typical aerobic APB of the order Sphingomonadales, whereas betaproteobacterial phototrophy was considerably reduced (Fig. 3). Interestingly, Rhodobacterales represented a significant portion not only of the total but also the active community (Figs. 3 and 4). A substantial part was represented by members related to the strictly aerobic family *Hyphomonadaceae* (OTU16) (Fig. 4). A closer inspection revealed that species related to the genus *Rhodobaca* (OTU69) were amongst the most active Rhodobacterales (Fig. 4). All the cultured *Rhodobaca* species are photoheterotrophic species originating from soda lakes^54^, but it seems that their freshwater relatives were present in Římov. We did not observe any sequences showing similarity to the genus *Rhodobacter*, which were reported in previous studies^22,26,44^. This documents that the local phototrophic community was formed by aerobic species. This is consistent with the fact that the Římov Reservoir has stable thermal stratification and long retention time (in summer approx. 3 months)^55^, which makes a random input of anaerobic species from the sediment or soils unlikely. As a result, the microbial community inhabiting the euphotic zone is almost exclusively planktonic and aerobic. Obviously, a different situation may occur in small or very shallow water bodies, where populations of aerobic and (mostly) anaerobic APB species may mix.

In conclusion, we showed that the contribution of individual APB phylotypes to total communities does not directly relate to their contribution to active phototrophic communities. In spite of the large diversity of phototrophic species, light repression of photosynthetic gene expression seems to be a common feature of all aerobic APB bacteria present in the studied lakes.

## Materials and Methods

### Study area and sampling

*Cep lake* is an oligotrophic lake situated in the Třeboň Basin Protected Landscape Area, Czech Republic. It originates from sand mining during 1970–80s. The lake has no inflow or outflow and it has been filled with groundwater penetrating from the nearby river Lužnice. The diel sampling campaign was conducted on the 16–18^th^ of August, 2016 through the course of 32 hours (two nights and one day).

*Římov Reservoir* is a meso(-eu)trophic canyon-shape reservoir in the southern Czech Republic. It was built as a drinking water reservoir in 1970s (for details see^55^). Since there is constant water movement in the reservoir, we conducted the second experiment a 200 L mesocosm, which was set up 24 h before the sampling. The sampling campaign was conducted on the 23–24^th^ of August, 2017, spanning 24 hours.

During the experiment, continuous measurement of lake water physical and chemical parameters (temperature, pH, oxygen concentration, chlorophyll concentration) was done using the EXO1 multi-parameter sonde (YSI Inc., Yellow Springs, USA) deployed permanently *in situ* at the 0.5 m depth. Underwater irradiance was recorded by a submersible cosine quantum probe deployed together with the YSI probe. A submersible fluorescence probe (FluoroProbe, bbe-Moldaence, Kiel, Germany) was used to determine phytoplankton community composition^56^ (for details see the Supplementary file). In all the cases, the time of sampling refers to the local (astronomical) time.

Water samples were collected every two hours from 0.5 m depth. At each sampling time, ten liters of water were collected using a Friedinger sampler, and transferred to a plastic container, which was pre-rinsed three times with the sampled water. The water was then transported to the shore within 10 min and immediately processed as described below. At each sampling time, water was processed for pigment analyses, bacterial microscopy counts and nucleic acid extraction. Bacterial productivity was measured in Cep lake two times per day (at 6:00 and 18:00), and six times per day in Římov (for details see the Supplementary file). Primary production was measured between 6:00–10:00, 10:00–14:00 and 14:00–18:00 only during the experiment at the Římov Reservoir (for details see the Supplementary file).

### Pigment analyses

2 L of water from Cep lake, or 1 L from the Římov Reservoir were filtered on site through GF/F glass fiber filters, 47 mm diameter (Whatman plc, UK) within 30 min using a manual vacuum pump. The filters were dried of excess water, folded, wrapped in aluminum foil, flash-frozen and stored in liquid nitrogen. The samples were transported to the laboratory, and processed within 12 h. Pigments were extracted from homogenized filters in 8 mL of 7:2 v/v acetone:methanol mixture^35^. Clear extracts were analyzed using a Prominence-i HPLC system (Shimadzu Inc., Japan). For details, see the Supplementary file.

### Total bacterial and APB enumeration

10 mL of collected water sample were fixed on site with sterile-filtered formaldehyde to the final concentration of 1%, transported to the laboratory and stored at 4 °C in the dark for <48 h. Half ml of the fixed water was filtered onto white 0.2 µm polycarbonate filters (Nucleopore, Whatman), air-dried and mounted on microscopic slides with an anti-fading glycerol mix containing 4′,6-diamidino-2-phenylindole (DAPI) at concentration of 1 µg mL^−1^ ^57^. They were stored at −20 °C until processed.

Total bacterial and APB abundance was determined using epifluorescence Zeiss Axio Imager.D2 microscope, as described in Cepáková *et al*.^35^. Minimum 10 microphotographs were taken for every sample under UV/blue emission/excitation channel for DAPI fluorescence (total bacteria), blue/red emission/excitation channel for autofluorescence from Chl *a* (algae and cyanobacteria), and white light/infrared emission/excitation channel for autofluorescence from BChl *a* (APB). Minimum 1500 DAPI-stained cells were counted manually in ZEN software. As some part of Chl a autofluorescence is also visible in infrared spectrum, only the cells that did not showed autofluorescence from Chl a were counted as APB bacteria.

### Nucleic acid extraction and reverse transcription

Water samples (1 L) for nucleic acid extraction were filtered on site immediately after the collection. The cells were collected onto 0.22 μm Sterivex filter units (Millipore-Merck, USA) using sterile plastic syringes. The filtration was conducted under ambient irradiance (in the light during the day, in the dark during the night), and finished within 15 min. Then, the units were sealed, flash-frozen in liquid nitrogen, and stored at −80 °C until extraction.

Nucleic acids were extracted from the filters under aseptic, RNA-, DNA- and nuclease-free conditions, following the protocol by Nercessian *et al*.^58^ (for details see the Supplementary file).

### Quantitative reverse transcription PCR (RT-qPCR)

Prior to reverse transcription, DNA was removed from extracted nucleic acids using the Turbo DNA-free kit (Ambion, Invitrogen) according to the manufacturer routine protocol: 2 U of DNase were added to 30 µl of nucleic acids and incubated at 37 °C for 30 min. DNase was removed by adding 2 µl of DNase Inactivation Reagent. The absence of DNA in RNA samples was subsequently confirmed with PCR without reverse transcriptase. cDNA was generated from 200 ng of total RNA using the SuperScript IV VILO Mastermix (Invitrogen), according to the manufacturer’s protocol, with elongated time for reverse transcription (30 min). The obtained cDNA was diluted 5× and used directly in qPCR, or stored at −80 °C.

*puf*M gene expression was quantified in a relative quantification assay by RT-qPCR. The proteobacterial RNA-polymerase β subunit gene (*rpo*B) was used as a housekeeping reference gene. Prior to quantification, RT-qPCR conditions were thoroughly optimized. The efficiencies for *puf*M and *rpo*B primers were similar: 97% for *puf*M (R2 = 0.9983) and 98% for *rpo*B (R2 = 0.9964) over 2 logs of initial cDNA concentration. RT-qPCR assays of Cep samples were performed in a Rotor-Gene 3000 thermocycler (Corbett Research, Australia) and assays of the Římov samples in a CFX Real-Time Detection System (BioRad, USA). Each reaction was performed in triplicate. A 20 µL reaction contained 1× PowerUp Sybr Green master mix (Applied Biosystems, USA), 500–1500 nM of primer and 2 μL of 5× diluted cDNA (for PCR conditions see Supplementary Table S1). Relative gene expression fold change was calculated by the comparative Ct method^59^. The normalized expression of the sample at 6:00 (Cep) or at 7:00 (Římov) was used as the reference time point.

### *rpo*B gene primer design

Proteobacteria-specific, broad-range (degenerate) *rpo*B primers were designed in this study to match the commonly used universal *puf*M primers^20^. Proteobacterial and non-Proteobacterial *rpo*B gene sequences were retrieved from the GenBank database, aligned with MUSCLE^60^, and the primers were designed manually to target conserved regions specific for Proteobacteria. The specificity of the primers was tested *in silico* with BLAST and at the FunGene’s *rpo*B gene repository (http://fungene.cme.msu.edu/)^61^, using the *probe match* search function. Then, the primers were tested with PCR using DNA or cDNA from cultured species. Proteobacterial strains were used as positive controls. The identity of PCR products was confirmed using Sanger sequencing. Cyanobacterial and Bacteroidetes strains served as negative controls with no visible amplification.

### DNA- and RNA-based Illumina amplicon sequencing

#### *puf*M sequence library

Two *puf*M libraries were prepared from each lake, one from the DNA template and one or two from the RNA template. RNA amplicon libraries were generated from night RNA samples at 2:00 (Cep) and at 4:00 (Římov). DNA amplicon libraries were made from samples taken at 18:00 (Cep) and 4:00 (Římov). Libraries were prepared using the universal *puf*M primers (191 bp)^19^. All PCR amplifications (for conditions see Supplementary Table S1) were performed in triplicate, which were pooled and gel purified using the kit Wizard SV Gel and PCR Clean-Up System (Promega, USA). The sequencing was performed using Illumina MiSeq platform (2 × 250 bp) at Genomics Core Facility, Universitat Pompeu Fabra, Barcelona, Spain.

Processing of the obtained *puf*M sequences was carried out in SEED2^62^: reads were joined using the fastq-join function with default settings, primers were cut off and sequences were filtered for mean sequence quality >30 and the correct length of the amplicon (144–146 bp). After initial filtering, processing continued at the nucleotide level, chimera check was performed by UPARSE^63^. OTUs were clustered at 94% sequence similarity, a threshold previously calculated for the *puf*M gene^64^ and commonly applied in previous works^16,65^. The most abundant sequences per OTUs were used as representatives of the phylogroup and were identified by blastn function built in SEED2. For Proteobacteria we decided to keep the standard nomenclature with five classes: Alpha-, Beta-, Gamma-, Delta- and Epsilonproteobacteria. For the alpha diversity estimates, *puf*M libraries were rarefied by random selection to 30 000 sequences (except for the Cep DNA library, which had total sequence count about 26 000).

#### 16S rRNA sequence library

16S rRNA libraries were prepared from RNA templates of one daylight and one night sample with aim to assess the effect of the diel cycle on the active bacterial community. Samples collected at 14:00 and 2:00 from Cep and at 12:00 and 4:00 from the Římov Reservoir were sequenced. 16S rRNA transcript libraries were prepared using universal primers targeting the V3-V4 region (approx. 450 bp)^66^ in the same procedure as above (for details see Supplementary Table S1) and sequenced at the same facility.

Initial processing of the obtained sequences was carried out in SEED2 the same way as described above. After initial quality and length filtering, further processing was carried out using Silva NGS online platform (https://www.arb-silva.de/ngs/), with OTU clustering threshold set at 98% similarity and other settings left at default (sequences were already pre-filtered for quality and length).

Raw unpaired sequence reads were submitted to the NCBI database under BioProject identification number PRJNA527007.

### PufM phylogenetic tree construction

PufM protein sequences were retrieved from the GenBank of the representatives of Proteobacteria (4 sequences), Chloroflexi (10 sequences) and WPS-2 candidate division (2 sequences available to date). PufM protein sequence alignment was done by ClustalW with default settings. Phylogenetic analysis was conducted in MEGA7^67^ by Maximum Likelihood method based on the Le_Gascuel_2008 substitution model^68^, using bootstrap method to test phylogeny with 100 replications. A discrete Gamma distribution was used to model evolutionary rate differences among sites (5 categories (+G, parameter = 1.3941)). All positions with less than 80% site coverage were eliminated. That is, fewer than 20% alignment gaps and missing data were allowed at any position. There were a total of 297 positions in the final dataset. Additionally, sequences of uncultured members of Chloroflexi retrieved from freshwaters, and phylogroups identified in our study were added to the alignment. Phylogenetic analysis was again performed the same way as described above with 25 sequences and 48 positions in the final dataset.

## Supplementary information

Supplementary information

Supplementary information

## Data Availability

Data produced during this study (*puf*M and 16S gene and transcript sequence reads) were deposited to the NCBI database under BioProject identification number PRJNA527007 and are accessible for the general public.
