# Supplementary material for: Diel changes and diversity of pufM expression in freshwater communities of anoxygenic phototrophic bacteria
Source: Sci Rep. 2019 Dec 10;9:18766. doi: 10.1038/s41598-019-55210-x (PMC6904477; doi:10.1038/s41598-019-55210-x)
Supplement: Supplementary file 2 — Supplementary information [file 41598_2019_55210_MOESM2_ESM.pdf]

SUPPLEMENTARY FILE

Diel changes and diversity of *puf*M expression in freshwater communities of anoxygenic phototrophic bacteria

Lívia Kolesár Fecskeová[1], Kasia Piwosz[1], Martina Hanusová[1], Jiří Nedoma[2], Petr Znachor[2], Michal Koblížek[1]

[1]Lab. of Anoxygenic Phototrophs, Centre Algatech, Institute of Microbiology of the Czech Academy of Sciences, 37981 Třeboň, Czech Republic

[2] Biology Centre of the Czech Academy of Sciences, Institute of Hydrobiology Na Sádkách 702/7, 370 05 České Budějovice, Czech Republic.

Supplementary methods

Phytoplankton community composition

A submersible fluorescence probe (FluoroProbe, bbe-Moldaence, Kiel, Germany) was used to measure chlorophyll *a* concentration in a detailed vertical profile at ~ 0.2 m intervals down to 20 m depth. According to the specific fluorescence spectra of distinct groups of phytoplankton, the probe permits differentiation of Cyanobacteria, Chromophytes + Dinoflagellates (a mixed group with diatoms being frequently the most important), Cryptophytes and Chlorophytes in mixed natural populations (56). The fluorescence probe was provided with original software (FluoroProbe 1.8.4, bbe Moldaence), which allowed the quantification of each phytoplankton group expressed in terms of the equivalent amount of chlorophyll *a* per liter of water.

Nucleic acid extraction

Total nucleic acids (RNA and DNA) were isolated from the filters according to the protocol described in Nercessian *et al*. (58). Briefly, cut filters and 0.2 g of each 0.01 mm, 0.1 mm and 1 mm zirconia-silica beads (BioSpec Products, USA) were suspended in 750 µl of extraction buffer (a mixture of equal volumes of 10% CTAB [cetyltrimethylammonium bromide] in 1.6 M NaCl and 0.2 M phosphate buffer, pH 8.0), to which 75 µl each of 10% sodium dodecyl sulfate and 10% lauroyl sarcosine were added. After addition of 750 µl of phenol-chloroform-isoamyl alcohol (25:24:1), the mixtures were homogenized in a minibeater (BioSpec Products, USA) for 3 min and then centrifuged at 16,000 × g for 5 min at 4°C. The aqueous phase was mixed with an equal volume of chloroform-isoamyl alcohol (24:1) and centrifuged at 16,000 × g for 5 min at 4°C. Nucleic acids were precipitated by adding 2 volumes of 30% PEG 6000 in 1.6 M NaCl for 2 h at 4°C. Nucleic acids were

recovered by centrifugation at 16,000 × g for 90 min at 4°C, washed with 75% ethanol, and resuspended in 30 µl of sterile nuclease-free water.

Pigment analyses

Pigments were extracted from homogenized filters (15 mL glass homogenizer and power-driven Teflon piston) in 8 mL of 7:2 v/v acetone:methanol mixture (35). Clear extracts were analyzed using a Prominence-i HPLC system (Shimadzu Inc., Japan) equipped with a UV-VIS diode-array detector. Pigments were separated using a heated (40°C) Phenomenex Luna 3µC8(2) 100 Å column with binary solvent system A: 20% 28 mM ammonium acetate + 80% methanol, B: 100% methanol, with flow rate of 0.8 mL min^{-1}. The peak for BChl *a* was registered at 770 nm and the pigment concentration in the original sample was calculated from the peak area. The HPLC system was calibrated using 100% methanol extracts of *Synechocystis* sp. PCC6803 and *Rhodobacter sphaeroides* with known concentrations of Chl *a* or BChl *a*, respectively.

Thymidine uptake by microbial community

Bacterial activity during the experiment at Cep lake was measured in samples collected at 6:00 and 18:00 of astronomical time. One liter of water was collected into a clean glass bottle and transported to the laboratory in cold box within 30 min. The assimilation rate of ^{3}H-thymidine (American Radiolabeled Chemicals, St. Luis, MO, USA) was measured in triplicate following the protocol by Kirchman *et al*. (ref. S1). 10 mL of water were aliquoted into 20 mL HDPE scintillation vials (Thermo Fisher Scientific, Waltham, MA, USA), and radiolabeled compounds were added to a final concentration of 20 nmol L^{-1}. Samples killed using trichloroacetic acid (TCA, final conc. 1%) served as negative controls. The vials were incubated at *in situ* temperature in the dark. The incubations were terminated after 1 h using TCA. The samples were filtered onto nitrate cellulose filters (pore size 0.2 µm, diameter 25

mm, Pragopor, Prague, Czechia), washed twice with 2.5 mL of ice-cold 5% TCA (5% solution, w/v) and subsequently twice with 2.5 mL of ice-cold 80% ethanol solution (v/v). The filters were air-dried, and dissolved in 1 mL of ethyl acetate. 4 mL the Ultima Golt LLT scintillation cocktail (Parkin Elmer, Waltham, MA, USA) were added, and samples were left for 48 h in the dark. The radioactivity in the samples was measured using a Tri-Carb 2810 TR scintillation counter (PerkinElmer Inc., Waltham, MA, USA).

Bacterial production during the experiment at the Římov Reservoir was also measured in samples collected at 6:00 and 18:00 of astronomical time as described in Straškrabová *et al.* (ref. S2). Subsamples (10 mL) of water sample were incubated with ^{3}H-thymidine (final concentration 20 nmol L^{-1}) at *in situ* temperature for 30 min. Incubations were terminated by the addition of 2% formaldehyde (final concentration, the same was used for blanks), samples were then filtered through 0.2 µm polycarbonate filters and extracted directly on filters with 2 mL of ice-cold 5% TCA 5 times for 1 min. The cell production rate (cells L^{-1} day^{-1}) was calculated assuming the theoretical conversion factor of 2×10^{18} cells per mol of thymidine.

Primary production

Primary production (PP) and extracellular release (ER) were measured with the ^{14}C method as described in Straškrabová (ref. S2). Water samples (two light and two dark bottles, volume cca 120 mL^{-1}) were incubated *in situ* at 0.5 m depth for 4 h. Each bottle received 0.1–0.2 MBq of carrier-free ^{14}C-bicarbonate. From each bottle, 1 mL aliquot of incubated sample was transferred to a scintillation vial containing 20 µl of 5M NaOH (to prevent a loss of ^{14}C-bicarbonate), and a 40 mL aliquot was filtered through a 1 µm polycarbonate filter (Poretics, USA, diameter 47 mm). The resulting filtrate contained dissolved ^{14}C-DOC plus ^{14}C-DOC taken up by bacteria during incubation. Very low vacuum of <0.01 MPa (<3

in Hg) and a filter funnel with a large filtration area (10.2 cm^2) were used to avoid cell breakage during filtration. Three parallel 10 mL portions of the filtrate were transferred to scintillation vials, acidified by adding 200 µL 5M HCl, and air-bubbled for 1 h to remove unassimilated inorganic ^{14}C. The total added, filter-retained and filtrate ^{14}C activities were measured after the addition of 10 mL of scintillation cocktail (Ultima Gold XR, Sigma-Aldrich, St Louis, USA) with scintillation spectrometry. Extracellular release (ER) was assumed to be equivalent to ^{14}C-DOC present in filtrate, primary production (PP) was calculated as the sum of filter-retained ^{14}C and ER. To get carbon fluxes (µgC L^{-1} h^{-1}), the rates (h^{-1}) (calculated as a fraction of the added inorganic ^{14}C incorporated or released per hour) were corrected for dark-bottle values and incubation time, and multiplied by the concentration of dissolved inorganic carbon (DIC), calculated from pH and the alkalinity determined by Gran titration.

Supplementary Table S1: Summary of primers used in this study and PCR/qPCR conditions

Primer	Sequence (5'→ 3')	Description	Conditions*	Product length	Reference
**pufM F**	TACGGSAACCTGTWCTAC	**RT-qPCR**<br>Universal primers for the reaction center subunit M	Fw 1000 nM<br>Rev 500 nM<br>58°C,	198 bp	20
**pufM R**	CCATSGTCCAGCGCCAGAA				
**rpoB F**	GARCARGAVGTBTAYATGGGBGA	**RT-qPCR**<br>Proteobacteria-specific RNA-polymerase sigma factor	Both primers<br>1500 nM<br>58°C,	197 bp	This study
**rpoB R**	CCANGARCCRCGRTVGGRAT				
**pufM F**	GGNAAYYTNTWYTAYAAYCCNTTYCA	**Amplicon sequencing**<br>Universal primers for the reaction center subunit M	Both primers<br>1500 nM<br>57°C,	191 bp	19
**pufM R**	YCCATNGTCCANCKCCARAA				
**16S F**	CCTACGGGNGGCWGCAG	**Amplicon sequencing**<br>V3-V4 region of the bacterial 16S gene	Both primers<br>500 nM<br>55°C,	460 bp	66
**16S R**	GACTACHVGGGTATCTAATCC				

*Final primer concentration and annealing temperature

Supplementary Table S2: Alpha diversity indices of *puf*M libraries rarefied by random selection to 30 000 sequences, except for the Cep DNA library, which has total number of sequences 26 225.

	Cep lake			***Římov Reservoir***	
	DNA	***RNA1***	***RNA2***	***DNA***	***RNA***
Total reads	26 225	30 000	30 000	30 000	30 000
Number of OTUs	323	297	360	423	325
Shannon-Wiener DI	3.42	3.32	3.35	3.83	3.26
Simpson DI	0.082	0.074	0.068	0.044	0.093
RICHNESS Chao-1	491	466	765	692	548

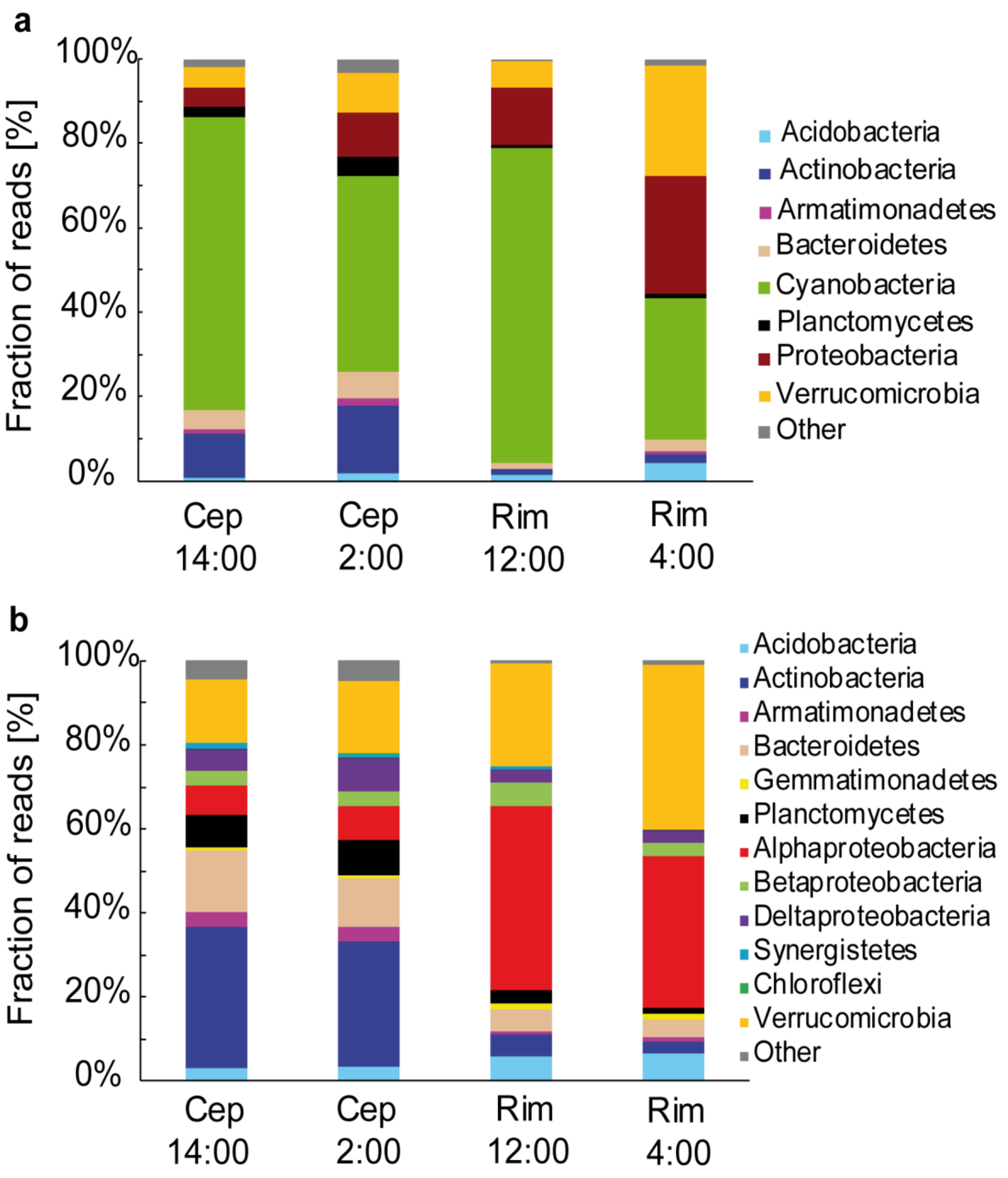

Supplementary Figure S1. Composition of total bacterial 16S rRNA transcript amplicon libraries of Cep lake (Cep) and the Římov Reservoir (Rim) in day (14:00 in Cep, 12:00 in Římov) and night (2:00 in Cep, 4:00 in Římov) samples. Relative abundance of the main phyla (a) and relative abundance of all phyla and subdivisons of Proteobacteria after subtraction of cyanobacterial reads (b).

17

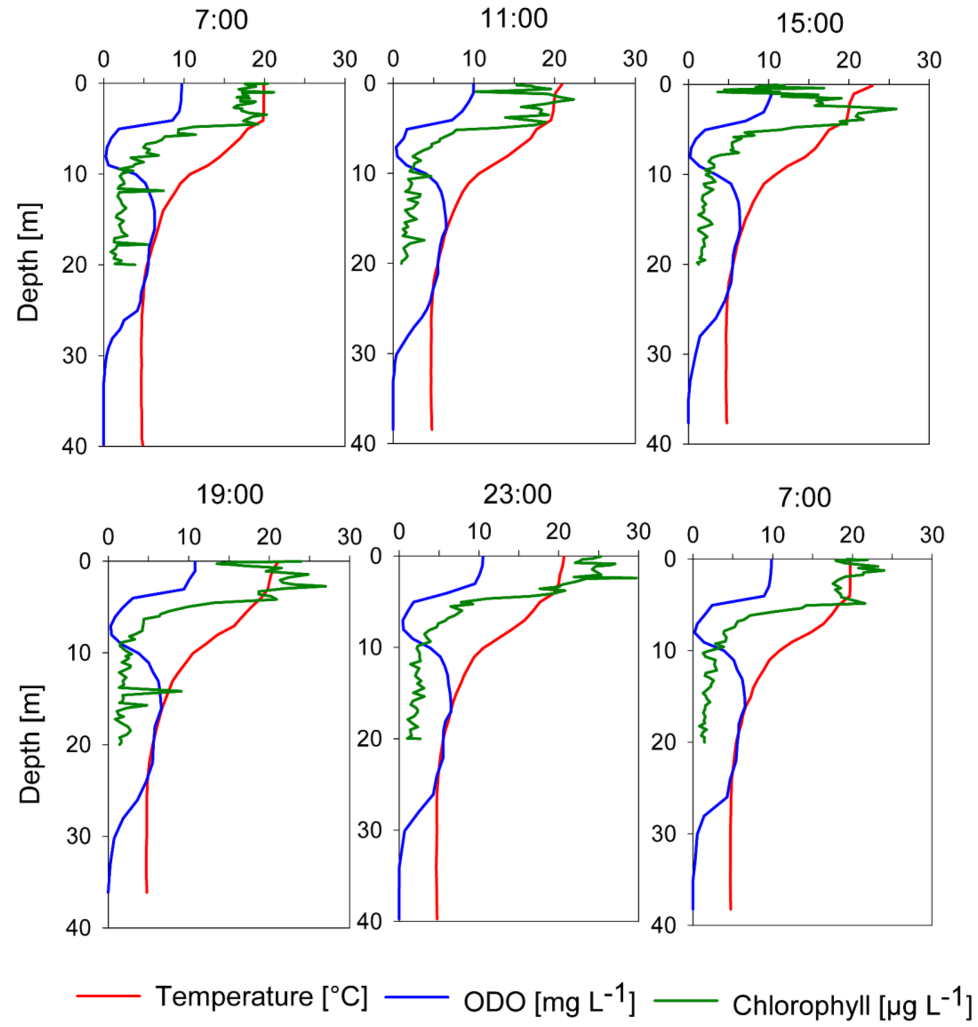

Supplementary Figure S2. Water vertical profiles of temperature, concentrations of dissolved oxygen and chlorophyll in the Římov Reservoir on August 23–24, 2017, measured at the dam in different times through one diel cycle.

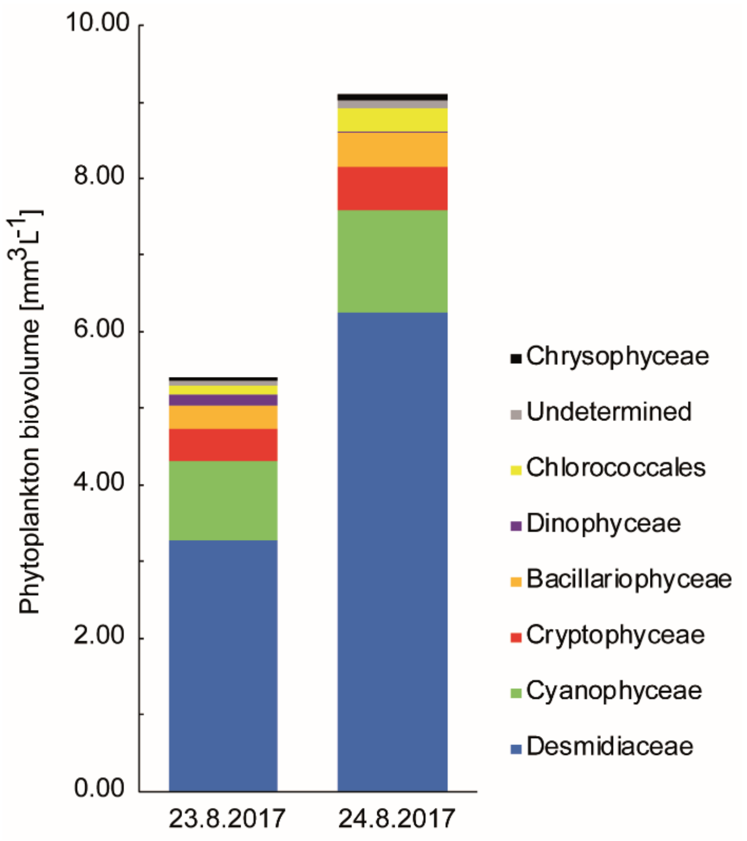

Supplementary Figure S3. Phytoplankton biovolume in the Římov Reservoir, determined in the mesocosm during the time of the experiment. Total biovolume was 5.41 $mm^3 L^{-1}$ and 9.11 $mm^3 L^{-1}$ on August 23rd and 24th, 2017, respectively.

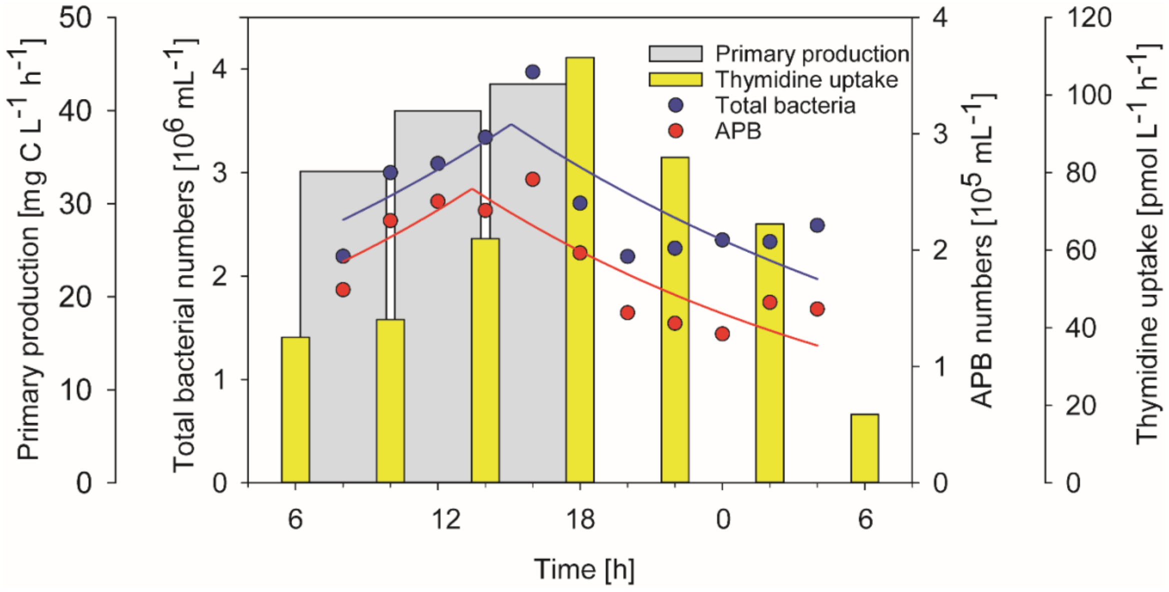

Supplementary Figure S4. Total and APB bacterial numbers, thymidine uptake and primary production in the Římov Reservoir, August 23–24, 2017. Primary production was measured between 6:00–10:00, 10:00–14:00 and 14:00–18:00.

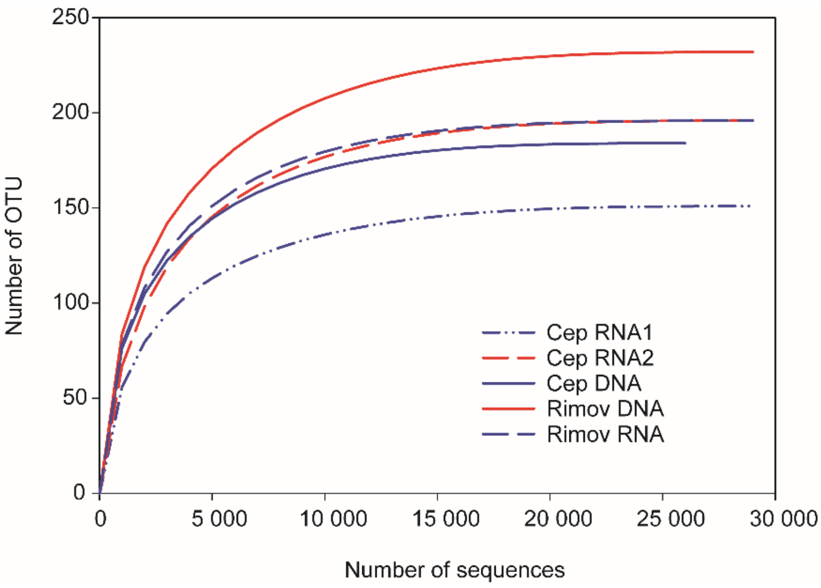

Supplementary Figure S5. Rarefaction curves at 94% OTU similarity. Cep DNA library contains 26 000 sequences, all the other libraries were rarefied to 30 000 sequences, after which singletons were excluded from the dataset.

Additional references

S1 Kirchman, D., Knees, E., Hodson, R. Leucine incorporation and its potential as a measure of protein-synthesis by bacteria in natural aquatic system. *Appl. Environ. Microbiol.* **49,** 599–607 (1985).

S2 Straškrabová, V. *et al*. Investigations on pelagic food webs in mountain lakes - aims and methods. *J. Limnol.* **58,** 77-87(1999).
